# Observation and experimental investigation of confinement effects on ion transport and electrokinetic flows at the microscale

**DOI:** 10.1038/srep37236

**Published:** 2016-11-17

**Authors:** Anne M. Benneker, Jeffery A. Wood, Peichun A. Tsai, Rob G. H. Lammertink

**Affiliations:** 1 Soft matter, Fluidics and Interfaces Group, Faculty of Science and Technology, University of Twente, 7500 AE Enschede, The Netherlands

## Abstract

Electrokinetic effects adjacent to charge-selective interfaces (CSI) have been experimentally investigated in microfluidic platforms in order to gain understanding on underlying phenomena of ion transport at elevated applied voltages. We experimentally investigate the influence of geometry and multiple array densities of the CSI on concentration and flow profiles in a microfluidic set-up using nanochannels as the CSI. Particle tracking obtained under chronoamperometric measurements show the development of vortices in the microchannel adjacent to the nanochannels. We found that the direction of the electric field and the potential drop inside the microchannel has a large influence on the ion transport through the interface, for example by inducing immediate wall electroosmotic flow. In microfluidic devices, the electric field may not be directed normal to the interface, which can result in an inefficient use of the CSI. Multiple vortices are observed adjacent to the CSI, growing in size and velocity as a function of time and dependent on their location in the microfluidic device. Local velocities inside the vortices are measured to be more than 1.5 mm/s. Vortex speed, as well as flow speed in the channel, are dependent on the geometry of the CSI and the distance from the electrode.

Ion selective membranes are important for a variety of applications, such as water desalination, energy harvesting and in biological systems^1^,^2^,^3^. Ion transport phenomena near membranes have been of interest for many decades and electrokinetic effects associated with ion selective membranes have been investigated widely in the recent years^4^,^5^,^6^,^7^. Due to the recent development of micro- and nanoscale fabrication methods, numerous microfluidic experiments on electrokinetic phenomena adjacent to charge-selective interfaces (CSI) have been conducted^8^,^9^. The advantages of microfluidic systems include the control of the imposed flow, which is mostly in the laminar regime, the ease of handling and the small volumes required for experimenting. Most importantly, the optical access and visualization techniques available with microfluidic systems are especially beneficial for the fundamental understanding of ion transport phenomena taking place on the microscopic interface in macroscopic systems.

Investigations of microfluidic charge-selective systems typically consist of two or more micro-scale channels which are interconnected through a charge-selective layer^10^,^11^,^12^. This charge-selective layer can be a classical membrane, such as Nafion^9^,^10^, or a medium providing charge selectivity through double layer overlap, such as nanochannels^13^,^14^. The driving voltage is applied through electrodes located relatively far from the membrane interface. Due to the many different geometries and materials employed as CSI, the electrical measurements typically cannot be compared to each other directly^11^,^13^,^15^,^16^. Observations of ion concentration polarization (ICP) regions using charged fluorescent dyes have yielded information on the size of ion depletion zones as function of applied voltage^13^ and the formation of multiple depletion zones in charged membrane systems^11^,^17^. Local electric field strengths have been measured using electrodes inserted in the depleted microchannel^18^, showing that the local electric field strength in the depletion zone is high as a result of the depletion of charge carriers. In a similar way, the local ion concentration was measured in a dead-end channel with a Nafion membrane^19^.

Fluid vortex formation has been both predicted from numerical research and experimentally observed in the overlimiting current regime (OLC) adjacent to a CSI^5^,^20^,^21^ for different geometries and applied electric fields. Many numerical studies have been executed on the prediction of these fluid flows, with different proposed explanations for the occurrence of the OLC regime^5^,^20^,^21^,^22^, depending on geometry and other system parameters. Experimentally, vortex structures and depletion zones associated with vortex-like rolls have been observed in multiple microfluidic systems^7^,^8^,^12^,^13^,^19^,^23^,^24^, using particles and dyes. These results have shown the presence of multiple vortices in the vicinity of the CSI, but not the formation of these vortices and their growth as function of time in detail for microfluidic systems. In large systems, the vortex size and speed grow in time, up to a velocity of 80 *μ*m/s and a size of 1000 *μ*m as a function applied current density^23^,^25^. These hydrodynamic vortices can be utilized for creating micromixers^22^,^26^,^27^ in Lab-on-a-Chip applications, as they enhance mixing in a laminar system.

Often, in microfluidic systems and numerics in particular, the assumption is made that the electric field is in the same overall direction as the ion transport through the charge-selective interface and driving over the entire domain^20^,^21^. The majority of experimental papers simplify these relationships of the local electric field variations and the difference between the applied and induced local electric fields to provide order of magnitude or scaling law interpretations^13^,^19^. However, the nature of most microfluidic systems does not necessarily provide such a normal electric field as a result of the distortion of electric field lines through the geometry of the microchannels. In numerical research, the applied field is also typically assumed to be normal to the CSI for numerical simplification. In this paper, we experimentally show that this electric field distribution has a significant contribution to the observed phenomena and membrane performance, by using different geometries of the charge-selective interface. Concentration profiles are obtained using fluorescent dyes and particle tracking experiments are done for observation of flow patterns and speeds inside the microchannels. These experiments show that the direction of the electric field and small distortions in this, have a large influence on the ion transport through and near charge-selective interfaces.

## Physical background

All solids placed in an electrolyte solution obtain a surface charge, which induces an electric potential distribution inside the liquid. Ions present in the solution will re-distribute according to this potential, yielding an electric double layer (EDL) adjacent to the surface. The size of the EDL is dependent on the ionic strength of the surrounding solution and becomes larger for lower ionic strengths. The typical size of the EDL is described by the Debye length, 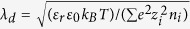, where *ε*_*r*_*ε*_0_ is the dielectric permittivity of the medium, *k*_*B*_ is Boltzmann’s constant, *T* is the temperature (in K), *e* the elementary charge, *z* is the ion valency and *n* is the concentration of ionic species (in mole/m^3^)^28^. If two walls are spaced in the order of this Debye length (O(10 nm) for 1 mM NaCl solution) or less, co-ions are largely excluded from the resulting channel due the overlapping EDL, resulting in selective ion-transport of counter-ions through this channel. An advantage of nanochannels over the use of classical membranes such as Nafion is their defined geometry and the controlability of the interface.

**Figure 1:**
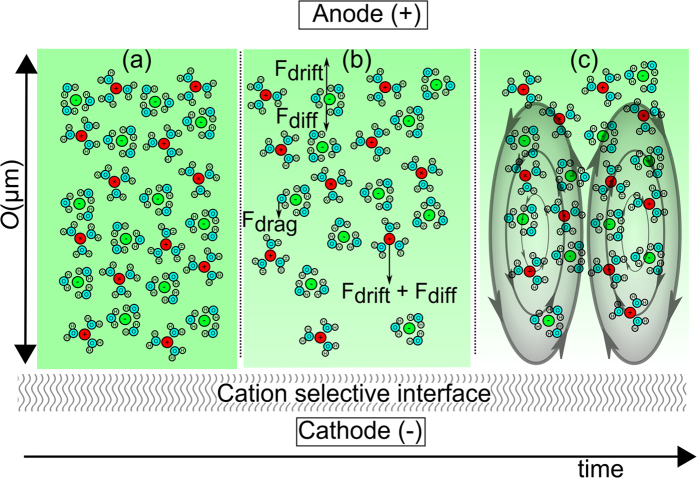
Schematic representation of a CSI in ionic solution under the application of an electric field. Depletion of both anions (green) and cations (red) at the interface occurs in the limiting current regime, yielding a transport limitation in the diffusive layer (indicated by the color gradient). Diffusive, drift and drag forces on the different ions and water result in fluid vortices as time proceeds. Before any potential or current is applied the ions are distributed evenly in the solution adjacent to the CSI (pane a). After the application of an electric field over the CSI an ion depletion zone is forming at the anodic side of the interface, while different forces are acting on the ions and water molecules in the solution (pane b). After proceeding of time, the combination of these forces (in combination with the absence of ions in the vicinity of the CSI) results in vortices forming adjacent to the CSI and within the boundary layer (pane c).

When an electric field is applied over a CSI in an ionic solution, counter-ions will migrate through the interface, towards their attracting electrode. Co-ions will drift towards the interface on the opposite side, but are blocked by the membrane, yielding a local increase in ion concentration at the interface and ICP over the membrane. Upon increase of the electric field, the depletion zone will grow into the solution and diffusion of ions through this zone will become the limiting step in the charge transport, yielding the well-known limiting current in the IV-curves^5^. For a cation-selective interface, the depletion zone will be on the anodic side of the interface and an enrichment zone is formed on the cathodic side. As a result of these concentration gradients and local potential gradients in the system, electroosmosis, (di)electrophoresis and diffusiophoresis will become more pronounced. The high local electric field strengths (as a result of low local ion concentrations) enhance these effects, yielding fast hydrodynamic developments inside the confined microchannel, as schematically depicted in Fig. 1. In this figure, as function of time, the development of hydrodynamic vortices is schematically shown. At *t* = 0 s, there are no concentration gradients in the system (pane a). Upon application of an electric field a concentration gradient forms (pane b), which results in diffusive forces acting on both cat- and anions. The applied electric field imposes a drift force on the cations towards the membrane. These forces cause movement of the ions, which in turn drag along the surrounding water molecules. These combined drift, diffusion and drag forces result in fluid movement in the vicinity of the CSI. Local concentration gradients result in tangential components of the electric field. As a result of these tangential components of the electric field in the electrically non-neutral parts of the ionic solution, a tangential force near the CSI arises and vortices are formed, shown in pane c of Fig. 1. These electro-hydrodynamic effects affect the diffusion limited layer adjacent to the CSI and contribute to the transition from the limiting towards the overlimiting regime in the IV-curve^29^.

Velocities of classical electroosmotic flows (EOF) are a function of the applied external field *E*_∞_, the dielectric permittivity of the medium *ε*_*r*_*ε*_0_, viscosity *η* and the wall zeta potential *ζ* and can be described by the Smoluchowski equation^30^, *u*_*eo*_ = −(*ε*_*r*_*ε*_0_/*η*) *ζE*_∞_. Velocities that are observed as a result of the concentration gradients are reported to be orders of magnitude higher than the predicted value for conventional wall EOF^18^,^13^.

## Experimental details

**Figure 2:**
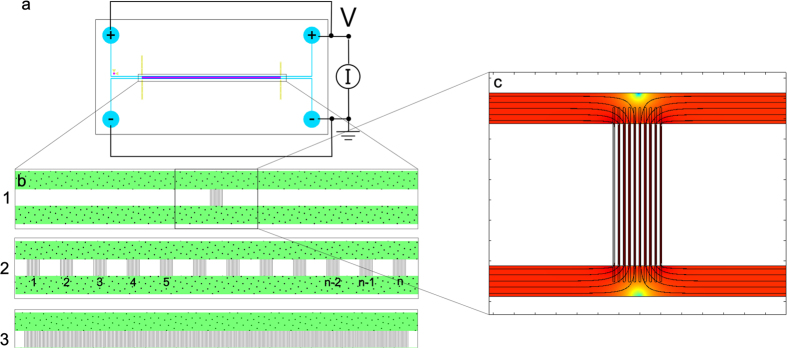
(**a**) Schematic design of micro/nano fluidic devices. Fluidic access holes are connecting the microchannels to liquid reservoirs on the chip holes. Potential is applied through silver electrodes that are placed in off-chip liquid reservoirs. (**b**) Two microchannels are connected through different distributions of nanochannels (1, 2 and 3). Patch numbers are indicated for type 2 chips. (**c**) Bending of electric field lines near the nanochannels in type 1 chips, simulated by COMSOL Multiphysics.

In our experiments, we used nanochannels (with a characteristic dimension of ~20 nm in depth) as charge selective medium in a salt solution of 0.1 or 1 mM NaCl, corresponding to a Debye length *λ*_*d*_ of 30 and 9.6 nm, respectively. Two microchannels (70 *μ*m in width, 20 *μ*m in depth) are connected through 50 *μ*m long nanochannels with a width and depth of 2 *μ*m and ~20 nm respectively, measured by white-light interferometry. Different distributions of nanochannels between the microchannels have been used, as shown in Fig. 2b, in (1) there is a single patch of 10 nanochannels (spaced 3 *μ*m), in (2) there are n = 50 of these patches (spaced 135 *μ*m) and in (3) there are 1660 nanochannels (all spaced 3 *μ*m). Chips are fabricated using wet etching of the nanochannels in glass, applying a sacrificial chrome-gold layer which is removed after wet etching of the microchannels. Access holes are powderblasted before thermally bonding two glass wafers. See Supplementary information for more details on the fabrication process. The glass has a negative surface charge, resulting in cation-selective nanochannels.

The microchips were placed in a dedicated chip holder and flushed with the desired salt solution for at least 30 minutes using a syringe pump, by first flushing one of the microchannels to make sure that all nanochannels are filled by capillary forces and afterwards flushing both channels. After flushing, the fluidic reservoirs in the chip holder were filled with the desired solution and the liquid was allowed to equilibrate inside the microchannels in the absence of any pressure gradients. To monitor ion concentrations, 0.1 mM NaCl solutions containing an additional 5 *μ*M Alexa Fluor 488 Cadaverine were used. For the visualisation of the flow fields, 0.05 wt% fluorescent polystyrene particles with a diameter of 1.0 *μ*m were added to the solution. Electric potential was applied by a Keithley 2450 Source Meter, controlled by LabVIEW. Silver electrodes were placed in all four fluidic reservoirs and a constant potential was applied to both electrodes connecting the upper microchannel while keeping the other two grounded, or vice versa. During these chronoamperometric measurements, resulting current and applied potential were recorded. Fixed potentials between 0 and 100 volts were applied for all geometries. Due to the presence of the charge-selective nanochannels the electric field lines are bent with respect to the channels. We have confirmed this by a simple COMSOL Multiphysics model of type 1 chips, from which we found the electric field lines to bend in the vicinity of the nanochannels (See Fig. 2c). Movies were recorded using a Zeiss Axiocam 105 Color (for fluorescent dyes) and a Hamamatsu ORCA-Flash4.0 LT (for particle tracking) camera, mounted on an inverted Zeiss Axiovert 40 MAT microscope.

## Results and Discussion

**Figure 3:**
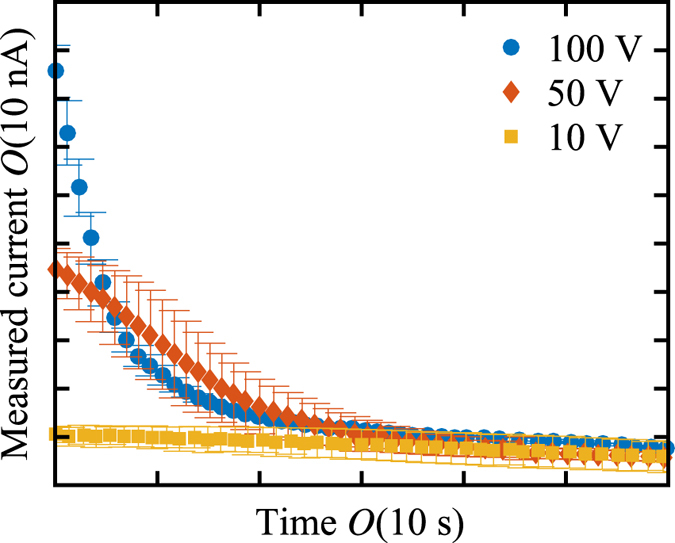
Typical chronoamperometric response for nanochannel chips as function of applied electric field strength. These results were obtained by a Type 2 (see Fig. 2) chip. Current decays as a function of time, for all applied potentials. Error bars are based on SD for multiple runs.

Typical chronoamperometric electrical responses of the different systems are shown in Fig. 3. A fast decay of current as function of time is observed for all configurations of nanochannels. This indicates that the ions available for transport become depleted from the CSI and that there is a strongly reduced transport of charged species through the nanochannels as time proceeds. Usually IV curves are reported, as opposed to chronoamperometric (*I* versus *t*) measurements^13^,^16^,^29^,^31^,^32^. When current as a function of time has been reported, researchers have observed similar decaying behavior for current as function of time for a fixed applied voltage^7^,^17^. The standard deviation (error bars) in measured current is in the order of ~20% relative standard deviation, which is in line with previous literature for nanochannels^33^.

In our experimental system the electric field is distorted as a result of the presence of the CSI (see Fig. 2c), yielding a growing resistance for ion transport as depletion zones are growing as is explained in the next section. An interesting note is the absence of an initial diffusive time scale, as electro-osmotic flows on the walls are present from the start of the measurements. The nanochannel conductivity and selectivity is extremely sensitive on its geometry. Due to the fabrication tolerance of our nanochannels, the quantitative electrical response for chips of the same type shows relatively large differences. However, the qualitative behavior as shown in Fig. 3 is found for all chips. On-chip reproducibility of the *I*-*V* and *I*-*t* data was relatively good, with a relative error of 4%, 20% and 46% for the measurements under 100, 50 and 10 volts respectively. The large error for the 10 V measurement is due to the relatively small currents (~nA). The fabrication tolerance of nanochannels is large as a result of the non-uniformity of the wet-etching process and cannot be measured quantitatively for all chips due to their small width. We measured the depth of a nanosized patch on different parts of the wafer before bonding, resulting in a difference in nanochannel depth of ~50% for different locations on the wafer. This results in a large deviation in area available for ion transport, which in turn yields large variations in the values of the measured currents if we compare different chips with the same geometry.

### Electric field distribution

Due to the location of the driving electrodes, the local potential driving the transport of ions through the nanochannels is much smaller than the applied potential. The electric field lines are locally distorted yielding that the electric field direction is not normal to the channels. This is a result of the nature of microfluidic systems, in which there are relatively long channels, which usually have corners that distort the flow and electric field, as stated before^18^,^27^,^29^. Therefore, the applied electric potential cannot be assumed to be dropped solely over the nanochannels.

**Figure 4:**
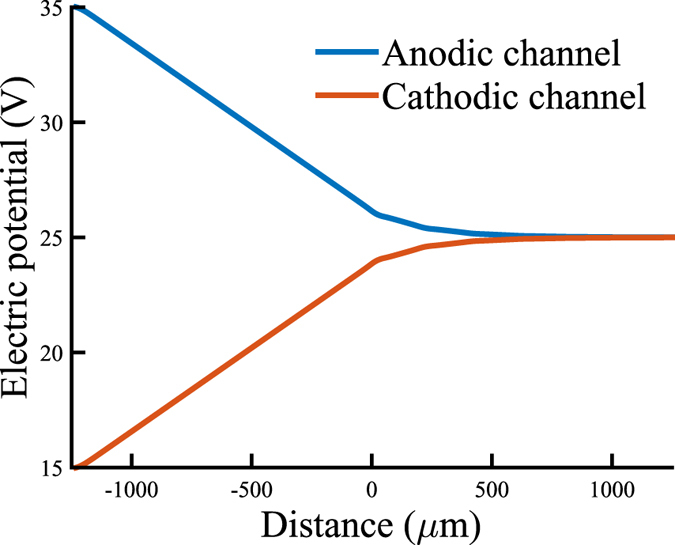
Local electric potential inside the microchannels of type 2 chips with an applied potential of 50 V in the anodic reservoirs, modeled in COMSOL Multiphysics. Distance 0 *μ*m is the position of the first nanochannel patch, in total 7 patches are represented in this figure.

For conformation of the proposed mechanism and the local electric fields, a simple quasi-3D model was build in COMSOL Multiphysics using the Electric Currents package. In Fig. 4 the resulting local electric potential in both the anodic and cathodic channel is shown. Before reaching the CSI, the local electric potential has reduced dramatically, yielding a relatively small driving force for ion transport through the CSI. The potential drop in the microchannel is a result of the length of the channels before the CSI is reached. It can easily be observed that the driving potential for the first patch is higher than for the second patch, and so on. From the seventh patch, the driving potential becomes negligibly small. This simplified model supports our hypothesis that the length of the microchannels has a large influence on the distribution of the electric field, which in its turn has a dramatic effect on the transport of ions through the nanochannels and the performance of the CSI. Placing the electrodes much closer to the CSI would reduce this effect, as has been noted for simpler geometries^29^. These findings are also in line with the recent work of Green *et al.*, who demonstrated the importance of the microchannel resistance for simpler microchannel/nanochannel geometries^34^.

### Concentration profiling

**Figure 5:**
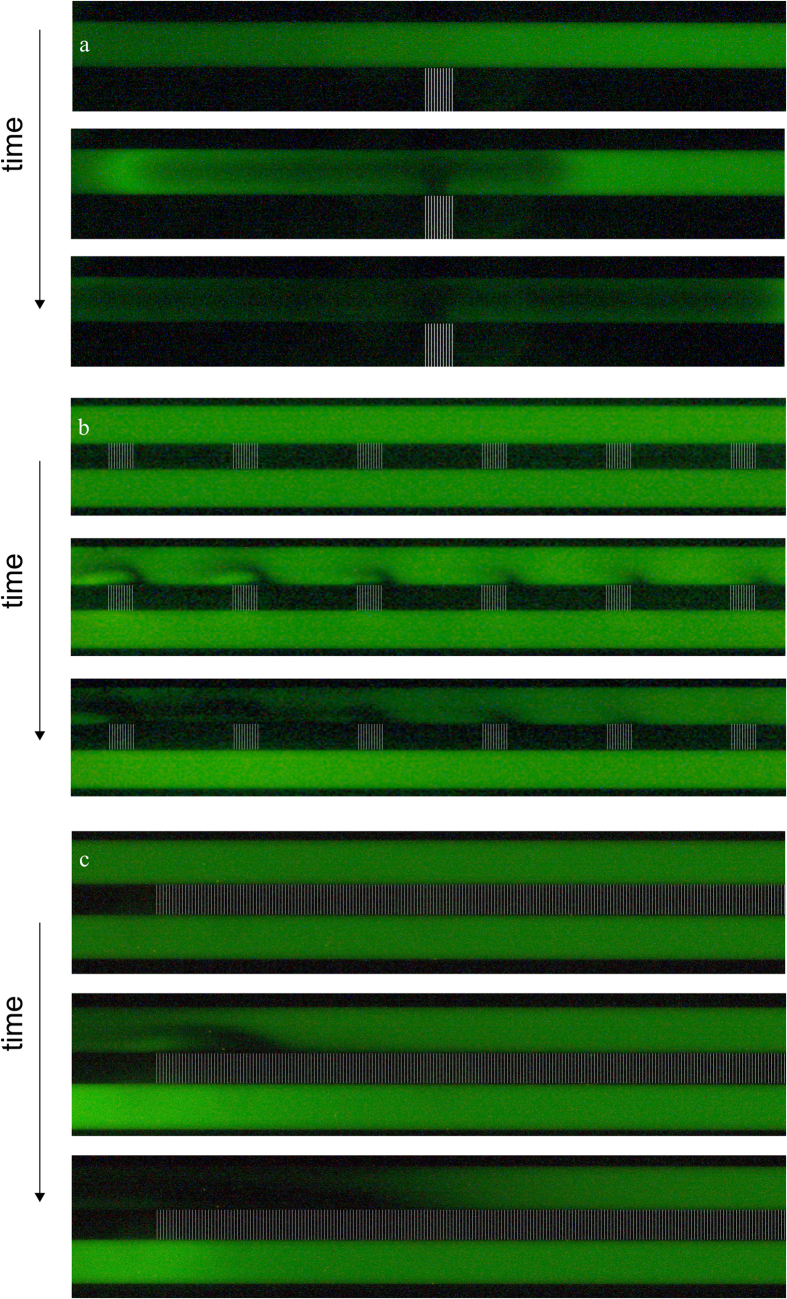
Intensity profiles of Alexa (negatively charged) for different chip geometries (geometry 1(**a**), 2(**b**) and 3(**c**)) upon the application of a driving potential of 50 volts. Both channels contain a 0.1 mM NaCl solution, seeded with 5 *μ*M Alexa fluorescent dye, the positive potential is applied in the upper channel for all geometries. Low intensity zones can be observed adjacent to the nanochannels in the anodic channel, indicating that locally the salt concentration is low.

To mimic the behavior of ions, a charged fluorescent dye is added to the salt solution. In our case, we used Alexa Fluor 488 Cadaverine (ex/em 490/525 nm), which has a negative charge and is pH independent in the measurement range (4 < pH < 10)^35^. Typical ion concentration profiles are presented in Fig. 5 for the three different geometries under investigation. For geometry 1 (a), we observe a gradual growth of the ion depletion zone on the anodic side of the CSI, similar to the observations of Kim *et al.*^13^. The size of the depletion zone grows as function of time and growth speed increases with increasing electric field strength. From this figure, it can be observed that the zone does not grow equally fast in both directions, which we attribute to a difference in local electric field, which might be the result of electrode placement, a slight pressure difference in the two reservoirs or bubble formation during the experiment. Results for type 2 chips are shown in Fig. 5b. The field of view contains the first arrays of nanochannels on the left. Wave patterns of ion depletion are developing in the vicinity of the patches of nanochannels, and growing in time until the depleted zone spans the entire width of the microchannel. The waves are forming as a result of the preferential direction of the transport of ions near the nanochannels and the interplay with the electric field which bends towards the nanochannels. It can be observed that the depletion patch above the patch closest to the electrode is faster and more intense than depletion above the patches further from the driving electrodes. This is a result of the local driving potential, which is drastically reduced along the microchannel towards the patches of nanochannels, as was shown in the previous section. The behavior observed is symmetric, as on the other end of the array of patches the same wave patterns are observed in a mirrored direction towards the other electrode (not shown in Fig. 5b, as a result of the limited field of view of our camera in comparison to the total geometry, but was visually observed to be symmetric). This results in a plug of ionic solution being trapped between two zones depleted of ions. For chips of type 3, only one depletion wave was formed at each end of the CSI, see Fig. 5c. In this system, symmetric behavior was also observed (again not shown due to the limitid field of view of our camera), resulting again in a trapped plug of ion-containing solution above the CSI, which seems unaffected by the imposed electric field. This indicates that the potential drop is located over just a portion of nanochannel patches and microchannel before the arrays, leaving most of the CSI unused in the transport of ions as there is no driving force for the transport. We note that this is likely the result of the local perpendicular direction of the electric field, which can also occur in larger membrane systems as a result of a small perturbation in the membrane surface^5^.

#### Influence of nanochannel configuration

**Figure 6:**
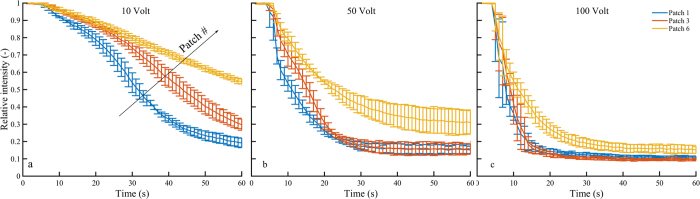
Dye intensity profiles (that mimic the local ion concentration) above different patches as function of time, for applied potentials of 10 V (**a**), 50 V (**b**) and 100 V (**c**). Patch 1 is the first patch from the electrode (as indicated in Fig. 2, patch 6 is the sixth. We averaged the dye intensity above the different patches as a function of time from different experimental runs, to obtain these profiles.

Using the type 2 chips, we see clear local differences in dye intensity and its decrease as function of time and applied voltage for the different patches of nanochannels. In Fig. 6, the average intensity of the dye in the channel above three patches in the field of view is plotted as function of time for three different applied voltages. Some additional information on this plots and the formation of these graphs is given in the Supplementary information. The patch closest to the driving electrode (patch 1, also indicated in Fig. 2) shows the fastest decay in local intensity, while the patch furthest from the electrode (patch 6) shows a significantly slower decay of intensity. A clear difference can also be observed for the different applied potentials, showing that for an applied voltage of 10 and 50 volts, the patches further from the electrode are not completely depleted of dye at higher times. This reduced dye intensity indicates a lower local ion concentration above patches closer to the electrode, resulting in a higher local electric field strength. Similar behavior is observed for chips of type 3, in which a depletion zone is forming on both sides of the array of nanochannels, but a plug of ion containing solution is trapped above the center of the CSI.

In our system, the electric field lines are curved significantly in the vicinity of the CSI, because of the geometry of the microchips. As a result of this and the local concentration differences, the local electric field strength and the potential drop over the nanochannels is not constant for all patches. We hypothesize that the potential drop over the patches further from the driving electrodes is significantly lower than the potential drop over the first patches. This results in a reduction of ion transport through the channels, as there is a small driving electric potential remaining for the charge transport. As a result, the local concentration above the patches in the center of the chip stays higher compared to the local concentration above the outer patches. Experimentally we observe that this effect is more significant for lower applied electric fields and that for higher applied electric fields a larger number of patches of show transportation of ions. For an applied voltage of 100 volts, all patches in the field of view are depleted from dye, although further from the electrode dye is still present above the patches. The electric potential is reduced across all patches, showing that for a higher starting potential, more patches are affected by the electric field and play a role in ion transport. This can also explain the reduction in measured current, as was shown in Fig. 3, which flattens out after the depletion zones have been fully established as there is a constant diffusive supply of ions towards the patches that are still influenced by the electric field.

#### pH effects

**Figure 7:**
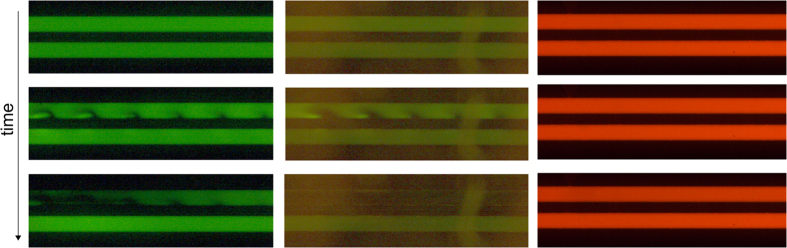
Pattern formation under the application of a 50 V electric field type 2 chips, with Alexa (left), Fluorescein (middle) and Rhodamine B (right) as indicative dyes for ion concentration and pH gradients. Alexa and Fluorescein are negatively charged, while Rhodamine B is uncharged. Fluorescein and Rhodamine B are known to be pH dependent in the measurement range, while Alexa is pH independent. Similar wave patterns are formed for both negative dyes, while no changes are observed in the intensity of the Rhodamine B experiments. This is an indication that no significant pH effects are influencing our measurements at this scale.

One of the possible mechanisms for overlimiting current is the formation of additional charge carriers, as a result of water splitting in the vicinity of the CSI^6^. This would yield protons and hydroxyl ions. To test if a pH gradient near nanochannels is developed in the system, we repeated the experiments with Fluorescein (495/517 nm), which is negatively charged and pH dependent and Rhodamine B (543/565 nm) which has no to a very slight charge, and is pH dependent in our measurement range. As can be observed from Fig. 7, the quantitative behavior of the Alexa and Fluorescein (both negatively charged) dye is similar, while for Rhodamine B (no charge) no gradients in intensity are observed. This indicates that the observed effects for the Alexa dye are a result of the negative charge of the fluorophore, and thus can serve as an indication of the local ion concentration. The Rhodamine B experiments did not show any alteration in intensity upon application of an electric field, which, together with the Fluorescein measurements can lead to the conclusion that there are no significant pH changes in the vicinity of the CSI in our system. This indicates that water splitting is not significantly occurring in this system, and no additional charge carriers are produced near the nanochannels under these conditions.

### Fluid flow and vortex dynamics

To be able to follow the fluid flow profiles inside the microchannels, fluorescent particles are seeded in the feed solution. Movies in which the flow is clearly visualized for the three different geometries can be found in the ESI. Typical frame rates for the capturing of the particle movement are between 100 and 150 fps, which at higher times and developed flow is too low for taking clear particle images. These frame rates are the highest possible for the system, as a result of light intensity required to maintain an acceptable image quality. This prohibits automatized particle tracking or PIV, but particles can be tracked manually. In all experiments no external pressure was applied, yielding no flows before an electric potential was applied.

For geometry 1, we observe two fast developing vortices in the anodic channel upon the application of the electric potential of 50 V. The vortices at first instance grow into the channel, but are being pressed onto the microchannel bottom walls at later times, which can be observed from Movie 1, available online. The size and speed of the vortices is growing as a function of time, just as the depletion zone was growing as function of time. Electro-osmotic instabilities (EOI) occur as a result of the extended space charge near the nanochannels (due to the concentration polarization) and the action of the non-uniform electric field on these regions^20^. The observed effect is an interplay between electroosmotic flows near the walls, electroosmotic instabilities in the vicinity of the nanochannel and dielectrophoretic/diffusiophoretic forces.

**Figure 8:**
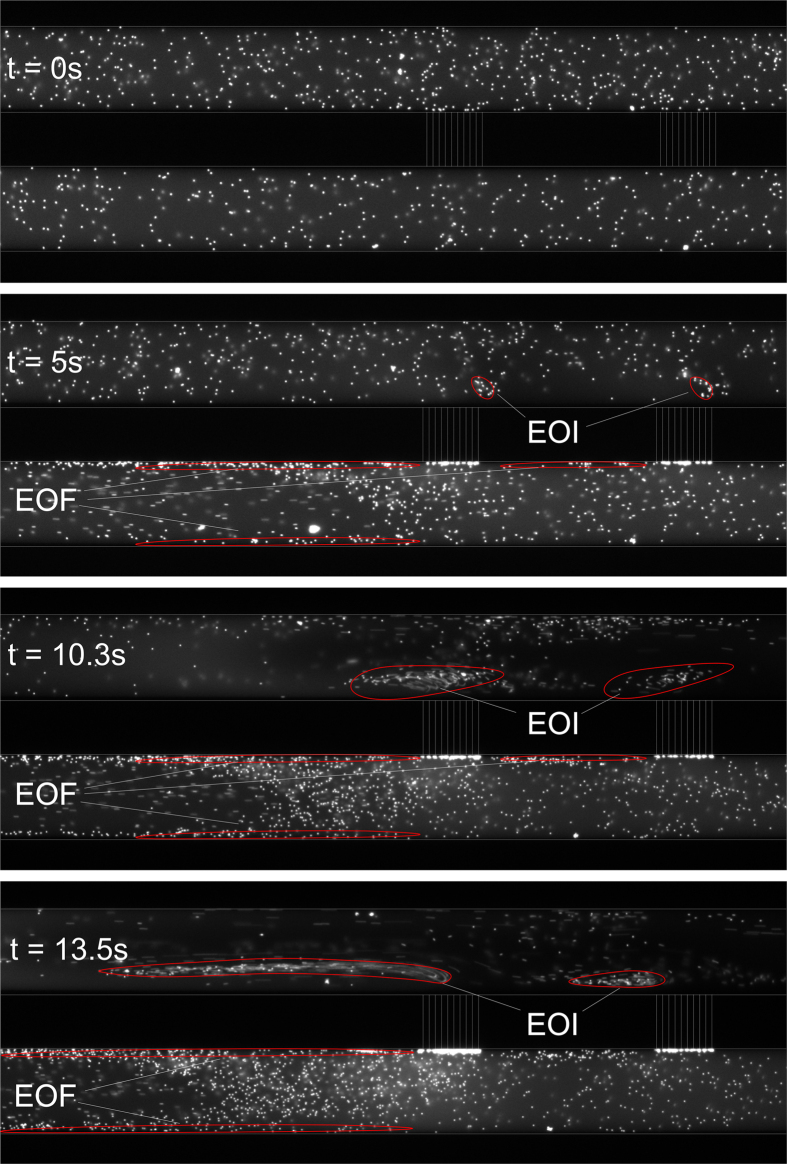
Fluid flow development over time for type 2 chips. Only patch 1 and 2 are in the field of view. vortices are observed above the different patches, and are growing in speed and size as a function of time. Regions of electro-osmotic flow (EOF) and electro-osmotic instabilities (EOI) are indicated.

In chips of type 2, vortices are observed in the anodic channel above several patches as can be observed in Movie 2 and Fig. 8. Upon application of the electric field, Movie 2 shows the formation of vortices in the anodic channel above five patches under the application of a 50 V potential, where the right end of the nanochannel arrays is imaged, patch n-5 to patch n. The vortex on the n-th patch (closest to the electrode on the right side of the channel) shows the fastest development and the largest final speed and size. This vortex extends significantly in the channel towards the driving electrode, while the vortices on the other patches stay relatively small and adjacent to their nanochannel patch. The vortices that are formed show a suppression towards the lower microchannel wall, just as type 1 chips. In the cathodic channel, movement of particles towards the nanochannels is observed, where they are retained by size exclusion forming particle cakes on the microchannel wall. At the walls between the patches particle movement in the direction of the expected EOF for a negative glass surface is observed. The chip shows symmetric behaviour, as was the case for the concentration profiles, on the other side (above patch 1 to 5) also vortices have been observed directed towards the other electrode. Vortex speeds and sizes grow as a function of time and applied voltage. This is also true for the observations in experiments with type 3 chips, in which a single vortex is growing on each end of the CSI in the anodic channel (see Movie 3 in ESI for one side of the CSI). More generally, these experiments indicate that a (small) distortion of the electric field and the magnitude of the residual field strength in membrane systems has a high influence on the efficiency of the membrane. A small distortion in this field can result in reduced capacity of the total membrane as part of the membrane is not contributing to the transport of ions.

The total fluid movement in the different geometries is a result of different forces acting on the ions, particles and liquid present in the system. EOF is present in both the anodic and cathodic channel, but is countered by different induced flows in both channels. In the anodic channel, the EOF becomes negligibly small in comparison to the flow as a result of the local non-electroneutrality in the depletion zones. We observe no net flow in the expected direction of EOF in the anodic channel in our experiments. In the cathodic channel, the EOF is countered by the electrophoretic (EP) mobility of the particles and ions inside the flow. Flow in the direction of the EOF was experimentally observed near the channel walls, but in the center of the channel the flow is dominated by the EP flow, see the movies in the ESI.

#### Quantification of observed flows

As stated, the detailed analysis of the obtained particle tracking images is done manually. Images were loaded into MATLAB and particles were tracked by identifying one and following it over multiple (up to 1000) frames to get local velocities inside and outside the vortices. We track particle clusters, as these can be unambiguously identified in every frame because of their characteristic larger size than single particles. We should note that we can only measure the velocity in the x and y-plane, and not the velocity in the z-direction. Here we only report speeds, which is defined as 
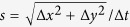
. The reported speed can be considered as a slight underestimation of the actual particle speed, because the z-component is neglected.

### Anodic channel

In type 1 chips, two vortices are formed, which are confined towards the lower microchannel wall and span approximately half of the microchannel width. The speed measured in these vortices is increasing with increasing electric field and time from application of this field. Maximium speeds inside the vortices are ~600 *μ*m/s, ~900 *μ*m/s and ~1000 *μ*m/s for applied electric fields of 20, 40 and 50 V respectively. These speeds are much larger than the flow speeds that would arise as a result of pure Smoluchowski EOF and similar to estimations by Kim *et al.*^13^. Inside the vortices the flow is not dominated by EOF, but by EOI.

For type 2 chips, the vortex speed for the vortex closest to the driving electrodes is measured to be ~500 *μ*m/s for a 10 V potential difference and ~1500 *μ*m/s for 50 V. The second patch has a significantly lower vortex speed, around ~600 *μ*m/s for an applied voltage of 50 V. The speed decreased for the patches further from the electrode, as can also be observed in Movie 2. This again indicates that the patches further from the electrode are not contributing as much in the phenomena taking place as the patches closest to the electrode. The absence of vortices above patches in the center of the microfluidic device also indicate that no significant transport is taking place there and thus that the entire CSI is not used optimally.

Flow speed in the channel above the suppressed vortices (in type 2 chips) is also dependent on the distance from the electrode. Particles are accelerated significantly as they cross the vortices from subsequent patches towards the driving electrodes. The measured speed of the fluid above the vortex on patch 3 is ~150 *μ*m/s, above patch 2 this speeds up to ~575 *μ*m/s and from the first patch on towards the electrode the speed is increased to ~1125 *μ*m/s. The speed of the flow on the anodic channel wall which is not covered by the vortex, so the upper wall, is measured to be ~200 *μ*m/s, also in the direction of the driving electrode. The flow in the anodic channel opposes the expected direction of EOF in the channel, indicating that the EOF is negligibly small in the anodic channel as a result of electrostatic forces on the extended space charge and to a lesser extent electrophoretic forces in this channel.

### Cathodic channel

The flow profile in the cathodic channel shows typical behavior of EOF in combination with EP. The speed on the channel walls is directed towards the grounded electrodes, in the expected direction for EOF, while the flow in the center of the channel is directed away from the electrode and towards the CSI, in the expected direction for EP.

The speed of the flow on the side wall was measured to be ~120 *μ*m/s in the direction of the EOF before the first patch for an applied electric potential of 50 V. Between the patches the speed on the wall is ~50 *μ*m/s. These speeds significantly reduce when the vortices are at full speed in the anodic channel. The speed in the center of the channel was measured to be ~200 *μ*m/s, in the direction opposing the EOF before the first patch, reducing to ~80 *μ*m/s after the second patch. This speed decreased with increasing distance from the electrode.

The maximum EOF was calculated, using the Smoluchowski equation, to be ~400 *μ*m/s, using a *ζ*-potential of −30 mV^36^ and an electric field strength of 20 kV/m (which is the maximum applied field strength). The electrophoretic mobility of particles with a relatively small electric double layer can be calculated using Hückel’s equation^30^, *μ* = *ζε*/(6*πη*), which in combination with electric field strength of 20 kV/m gives a predicted EP velocity of the particles of ~37–55 *μ*m/s if we assume the *ζ*-potential of the particles to be between −75 and −50 mV^28^,^37^. If EOF would only be countered by EP, the net direction of the particles would still be towards the electrodes, which is not observed experimentally. However, due to the symmetry of the chips and continuity, we expect a pressure-driven flow in the direction opposing the EOF on the walls^38^, which adds to the movement of fluid towards the nanochannel patches. We attribute the observed particle speed in the center of the channel to a combination of EP and EOF driven flows, resulting in a velocity that is higher than the single EOF and EP velocities. On the walls, the EOF (in the direction of the electrodes) is countered by the EP of the particles towards the nanochannels, which yield an experimental underestimation of the pure EOF on the wall (in the order of magnitude of the EP velocity of the particles). The order of magnitudes for these predicted flows are comparable to the order of magnitudes that we measured experimentally.

## Conclusion

In this paper, we have shown that the local electric field strength inside a microchannel has a large influence on the efficiency of charge-selective interfaces between these channels. Due to the nature of most microfluidic systems, the electric field is not necessarily distributed normally with respect to the CSI, yielding strong localized potential gradients and concentration gradients. The resulting electrokinetic instabilities are largely influenced by the drop in field strength with increasing distance from the electrodes. Results show that much of the membrane for our microchannel/nanochannel array system is not used efficiently, due to a combination of local distortions in the electric field as well as significant potential drop in the microchannel. These local distortions in the field and relatively large distance of the electrodes to the CSI are potential pitfalls inherent to microfluidic devices that should be considered when interpreting results. Small distortions yield a tangential wall component of the electric field to arise, resulting in different directions of EOF in the system. Large differences arise in fluid velocities above the different patches, the fastest vortices are observed closer to the electrodes. For macroscopic membrane systems, these problems may be less pronounced but distortions in the applied electric field could potentially have a strong influence on the membrane performance.

In the investigated nanochannel systems, local pH changes near the nanochannel entrances have not been observed which indicates that the vortex mechanism is entirely based on salt exclusion from the nanochannel arrays. Fluid movement is observed adjacent to the CSI in a limiting current regime in the depletion zones forming above the membrane. Flow speeds of *O*(mm/s) have been observed, both in vortices and the flows adjacent to the developing vortices. Large differences in the vortex speed and dynamics were observed between the various geometries, further highlighting the importance of the interplay of geometry and electrokinetic effects at the microscale.

## Additional Information

**How to cite this article**: Benneker, A. M. *et al.* Observation and experimental investigation of confinement effects on ion transport and electrokinetic flows at the microscale. *Sci. Rep.* **6**, 37236; doi: 10.1038/srep37236 (2016).

**Publisher’s note**: Springer Nature remains neutral with regard to jurisdictional claims in published maps and institutional affiliations.

## Supplementary Material

Supplementary Information

Supplementary Movie 1

Supplementary Movie 2

Supplementary Movie 3

Supplementary Movie 4
