# Supplementary material for: Observation and experimental investigation of confinement effects on ion transport and electrokinetic flows at the microscale
Source: Sci Rep. 2016 Nov 17;6:37236. doi: 10.1038/srep37236 (PMC5112581; doi:10.1038/srep37236)
Supplement: Supplementary Information [file srep37236-s1.pdf]

Observation and experimental investigation of confinement effects on ion transport and electrokinetic flows at the microscale

Anne M. Benneker, Jeffery A. Wood, Peichun A. Tsai, Rob G.H. Lammertink

Electronic supplementary information

Fabrication of micro/nanochannel chips

For the fabrication of the chips used in the experiments we cleaned glass wafers in HNO_3 before priming them with HMDS. To be able to align the nanochannels with the microchannels, we first etched alignment marks on the blank wafers before etching the nanochannels. Standard photolithography was applied for the marks, and the backside of the wafer was protected with foil before glass etching in BHF for 8 minutes. After stripping of the resist wafers were cleaned in HNO_3 again before applying the photolithography for the nanochannels. Nanochannels were etched for 30 seconds in BHF, after which the resist was stripped and the dimensions of the nanochannels were measured using a White Light Interferometer (Bruker WLI Gontour GT-I). After cleaning the wafers in HNO_3 again and drying them on a hot plate of 150°C immediately a chrome (15 nm) and gold (150nm) layer was sputtered on the wafers. This layer prevents further etching of the nanochannels in the further process steps. After sputtering, immediately photolithography for the microchannels is applied on the wafer. Gold and chrome are removed from the microchannel locations using dedicated etchants and backside foil is applied again before glass etching. In a 25% BHF solution the microchannels are etched into the glass until they are 20 µm deep, which is indicated by size indicators elsewhere on the wafer.

For the powder blasting of via-holes a photosensitive foil is applied on the backside of the wafer. Access holes are aligned and the foil is developed after which holes can be powder blasted. The foil is removed in a solution of $CaCO_3$, placed in an ultrasonic bath. The wafer is completely cleaned and particles are removed in an ultrasonic bath using acetone. The sacrificial chrome-gold layer is removed using the dedicated etchants and the wafer is cleaned before pre-bonding it to a cleaned glass wafer. The wafers are thermally bonded overnight before they are diced.

S.Figure 1

In S.Figure 1, the vertical averaged normalized dye intensity along the anodic channel is plotted for different times. Local valleys in the intensity indicate the local low salt concentration adjacent to the nanochannel patches. Overall, the dye intensity decreases as function of time. This goes faster for the patches closest to the driving electrodes. At the final measurement time, the dye intensity has a gradient, depicted by the red line. Figure 5 in the main manuscript consists of the full time-series of the averaged intensities above the different patches, so the development of the curves in S.Figure 1 as a function of time.

Movie 1

Vortex formation in the anodic channel for Type 1 chips in an 50V applied electric potential. Two vortices are developing and folding onto the channel wall. The vortices grow in size and speed as function of time, and become depleted with the dye (light grey background) upon growth of the ion depletion zone. Some particles are dielectrically trapped above the nanochannel patch.

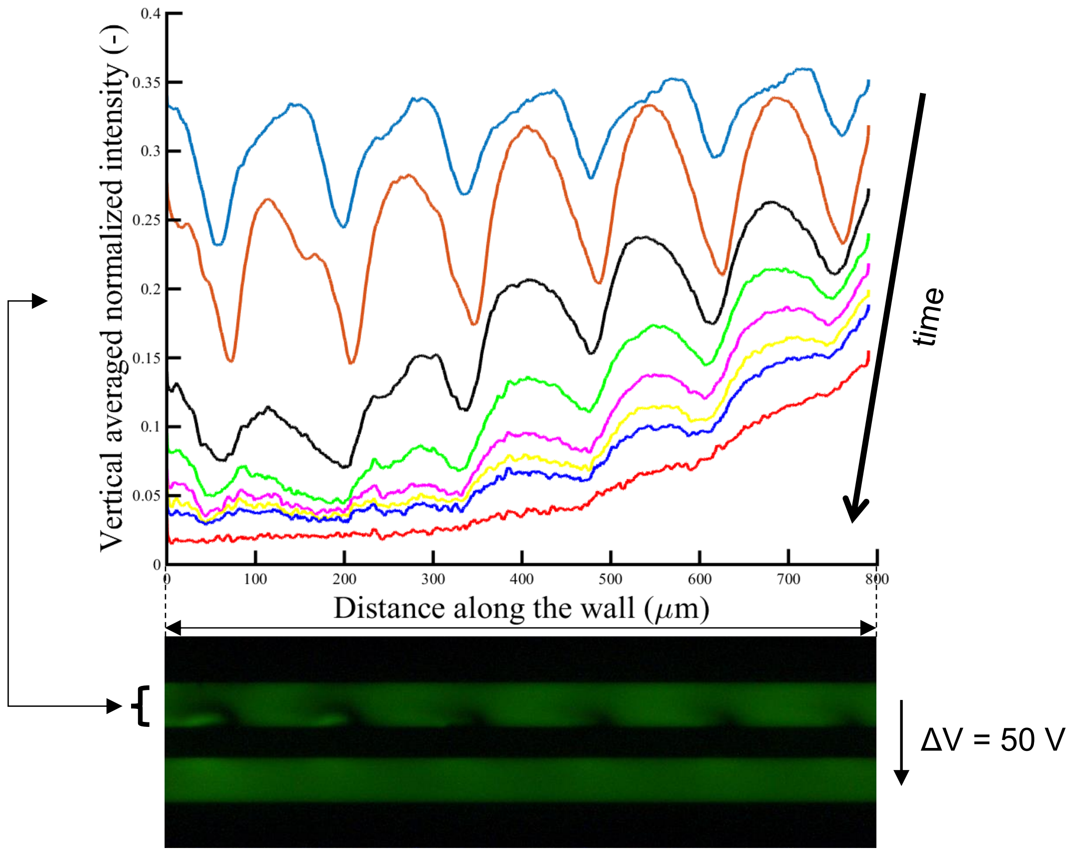

S.Figure 1 – Normalized intensity above patches as function of time and distance along the wall

<u>Movie 2</u>

Vortex formation in the anodic channel multiple patches of nanochannels in Type 2 chips under an applied electric field of 50V. Upon the start of the application of the electric field different vortices start to develop above the nanochannel patches. The patch closest to the electrode shows the highest increase of local speed, while vortices further from the electrode show much slower particle movement. The vortex closest to the electrode grows much bigger than the other vortices, which remain relatively constant in size after their development.

In the cathodic channel, the negatively charged particles move towards the nanochannels as a result of electrophoretic forces, where they are trapped because they are too big to pass the nanochannels. The particles on the wall show a slow movement towards the grounding electrodes in the direction of the expected EOF for positive ions that screen the negative microchannel walls.

<u>Movie 3</u>

Vortex development in type 3 chips. Upon the application of the electric field, particles move away from the interface in the anodic channel as a result of electrophoretic forces on the particles. A vortex forms above the nanochannels and increases in size and speed as function of time. The vortex moves towards

the edge of the CSI and spreads half of the anodic channel. In the cathodic channel, particles migrate towards the nanochannels, where they are blocked from transport.

Movie 4

Vortex formation in type 2 chips under the application of a 10V driving potential. Vortices appear above multiple patches, but their speed and size is much smaller than the vortices formed in Movie 2, under the application 50V. In the cathodic channel, the movement of EOF on the channel walls can be observed by the movement of the particles, but this is countered by the electrophoretic mobility of the particles.
